# Challenges and Innovations in Breast Cancer Screening in India: A Review of Epidemiological Trends and Diagnostic Strategies

**DOI:** 10.1155/ijbc/6845966

**Published:** 2024-11-28

**Authors:** Induni Nayodhara Weerarathna, Anurag Luharia, Ashish Uke, Gaurav Mishra

**Affiliations:** ^1^Department of Biomedical Sciences, School of Allied Health Sciences, Datta Meghe Institute of Higher Education and Research 442001, Wardha, Maharashtra, India; ^2^Department of Radio Physicist and Radio Safety, Datta Meghe Institute of Higher Education and Research 442001, Wardha, Maharashtra, India; ^3^Department of Radiation Oncology, Datta Meghe Institute of Higher Education and Research 442001, Wardha, Maharashtra, India; ^4^Department of Radiodiagnosis, Datta Meghe Institute of Higher Education and Research 442001, Wardha, Maharashtra, India

**Keywords:** breast cancer, cancer care, healthcare, India, navigating, oncology

## Abstract

The intricate terrain of breast cancer (BC) in India is examined in this review, which also looks at screening techniques, geographical differences, epidemiological trends, and obstacles to early diagnosis. BC has a major impact in India, especially on women. The research examines data from 2014 to 2024 and finds that, although overall cancer rates are declining, there has been a noticeable increase in BC cases. While obstacles including late-stage diagnosis and restricted access to treatment contribute to lower survival rates in India compared to Western countries, regional variations underscore the need for customized screening measures. The analysis of screening methods highlights the particular difficulties that Indian women encounter, such as the limitations of mammography in a country whose breast density is higher. The review presents cutting-edge technologies like breast exams and computer-aided detection and examines alternative techniques like ultrasonography. The importance of healthcare spending on screening uptake is highlighted by the regional inequality discussion, and mobile screening camps have emerged as a workable way to get around access and cost issues. The relevance of patient education and awareness in the Indian context is emphasized in the review's conclusion. The lack of adequate health resources and sociocultural obstacles, such as the fear of cancer, highlight the necessity of early detection campaigns and thorough education programs. With a knowledge of the difficulties and achievements in BC screening procedures, this narrative review hopes to make a significant contribution to the larger conversation about managing BC in the particular setting of India.

## 1. Introduction

### 1.1. Introduction and Epidemiology

Breast cancer (BC) is the most common malignancy in women worldwide [1]. Over time, there has been a decline in the incidence of BC in India. However, BC remains one of the most common tumors among women [2]. Epidemiological research indicates that, by 2030, there will likely be close to two million BC cases globally [3]. Between 1965 and 1985, the incidence in India increased dramatically by nearly 50%. The estimated number of incident BC cases in India in 2016 was 118,000 (95% uncertainly interval 107,000–130,000), 98.1% of which were in females, and the prevalent cases were 526,000 [4]. Over the last 26 years, from 1990 to 2016, the age-standardized incidence rate of BC in females increased by 39.1% (95% confidence interval, 5.1–85.5). BC had a cumulative risk of 2.81, accounting for 10.6% (90,408) of all deaths and 13.5% (178361) of all cancer cases in India, according to Globocan figures 2020 [5].

**Figure 1:**
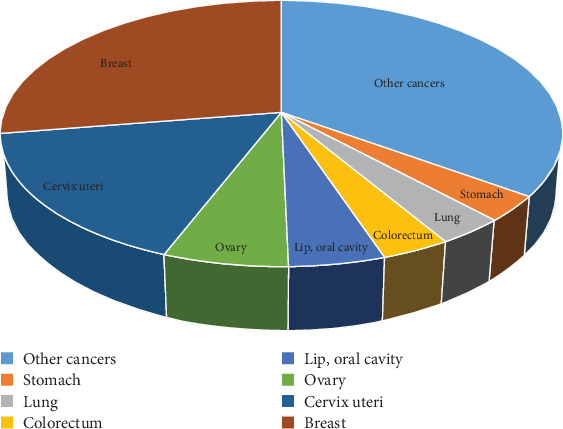
Estimated number of new cases in 2018, Indian females, all ages [6].

Figure 1 shows the number of women newly diagnosed with BC in 2018. In India, 162,468 women were diagnosed with BC. Furthermore, BC accounted for 27.7% of all newly detected malignancies in women. It essentially translates to one BC for every four newly discovered cases of cancer in Indian women.

**Figure 2:**
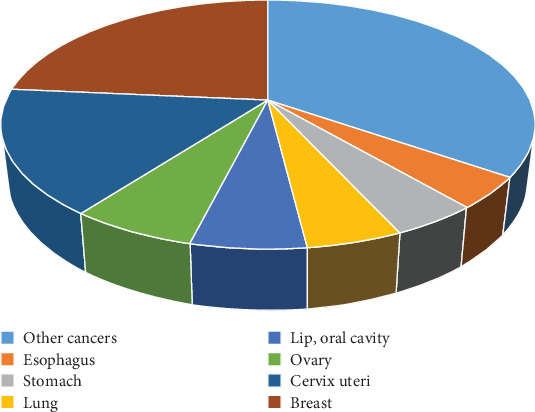
Estimated number of deaths in 2018, Indian females of all ages [6].

Figure 2 depicts the number of patients demised from a condition in a specific year which is referred to as “mortality rate.” In India, 87,090 women lost their lives to BC in 2018, according to Globocan. About 23.5% of all cancer-related deaths among Indian women were due to BC. This indicates that BC accounted for nearly one in four cancer-related fatalities among Indian women [6].

**Figure 3:**
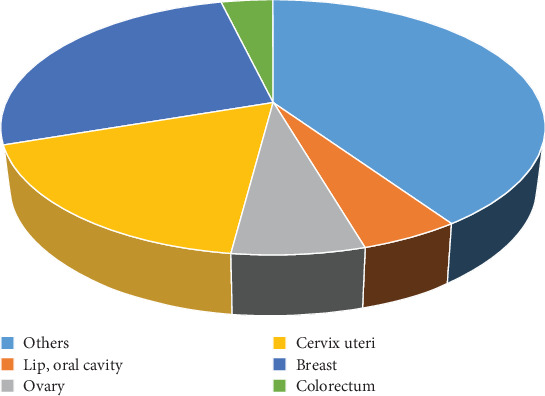
Number of new cases in 2022, females, all ages in India [6].

Current figures suggest that a higher number of Indian women are being affected by the disease at a younger age than women in the West. The National Cancer Registry Program examined information from cancer registries for the years 1988 through 2013 to monitor trends in the incidence of cancer. All population-based cancer registries have shown that the trend for BC has significantly increased [7]. Arumugham et al. [8] have conducted a survey. A total of 324 patients were included in the analysis. Among these patients, 60% had early BC (Stages I and II), 24% locally advanced and 4% had metastatic disease at presentation. A relatively high incidence of triple-negative cancers was seen (24%) while human epidermal growth factor receptor 2 (Her2)–positive disease was seen only in 8%. The 5-year overall survival for Stage I patients was 95%, that for Stage II was 92%, that for Stage III was 70%, and that for Stage IV was 21%. Her2-positive patients with early BC fared significantly worse when compared to luminal types (74% for Her2 type, 77% for triple-negative breast cancer (TNBC), 90% for Luminal A, and 99% for Luminal B cancers). India's BC survival rate is poorer than that of Western countries due to early onset of the disease, late stage at presentation, delayed initiation of definitive management, and inadequate or fragmented treatment [9]. Early identification and fast treatment are the most effective measures for controlling BC, according to the World Cancer Report 2020 [10].  Figure 3 shows the number of new cases in females, all ages, in India according to 2022 Globocan data.

The pie chart in Figure 3 illustrates the 2022 Globocan data on the incidence of cancer among women in India. According to the data, BC is the most prevalent type, representing 26.6% of total cases, followed by cervical cancer at 17.7%. These two cancers dominate the cancer landscape in Indian women. Ovarian cancer makes up 6.6%, while cancers of the lip and oral cavity contribute to 5% of the total cases. A significant portion, 37.1%, falls under the category of other cancers, which includes a diverse range of less common cancer types. This distribution highlights the critical public health need for focused cancer awareness, early screening, and treatment strategies, especially targeting breast and cervical cancers, which constitute a significant burden.

Although the effects of BC are felt throughout India, they differ from state to state. With projected cases, fatalities, and incidence rates for the entire country in 2020, Table 1 provides a window into the varied terrain of the illness. These numbers highlight the regional variations of this developing issue, from Kerala's alarming rate to Maharashtra's heavyweight. It is imperative to investigate these differences to guarantee that all women have equal access to diagnosis, treatment, and prevention as well as to tailored interventions [11].

BC screening is the process of assessing a woman's health for cancer problems before the onset of any disease-related symptoms or indicators. Every woman needs to be well informed and aware of her doctor's recommended cancer screening alternatives in India [13]. Together with their healthcare practitioner, this enables people to make informed decisions that help prevent and identify cancer-related problems early on. Greater chances of survival and recovery from cancer treatment can be achieved with early identification of the disease [14]. It is crucial to discuss the most appropriate BC screening tests with your physician. BC screening is crucial for all women, even those without symptoms, and involves a variety of tests, exams, and instruments. For early identification and prevention of BC, every woman must participate in a thorough breast screening program. The cancer's stage and degree of dissemination determine the prognosis. Cancer complications and metastases can be avoided with early identification of the disease [15]. This narrative review is aimed at comprehensively exploring the overview of BC screening in India. By examining the effectiveness of current programs, regional disparities, screening modalities and technologies, barriers to timely screening, and factors influencing patient compliance, the article is aimed at providing a nuanced understanding of the challenges and successes in BC screening practices.

## 2. Review Methodology

**Figure 4:**
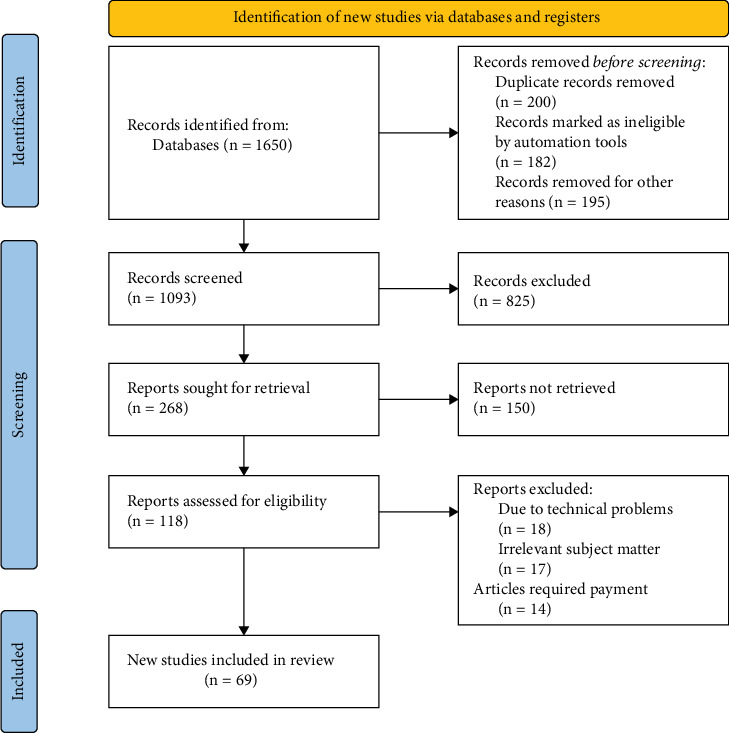
PRISMA flow diagram for search strategy and selection criteria (adapted from the preferred reporting systemic reviews and meta-analyses (PRISMA) guidelines).

A methodical approach to search strategy was used to find relevant materials for the narrative review “Navigating Breast Cancer in India.” We conducted a comprehensive search of academic databases, such as PubMed, Web of Science, and Scopus, to get pertinent research, reviews, and reports from 2014 to 2024. Particular keywords that were relevant to the review's subject matter were included in the search strategy, including “Breast Cancer,” “India,” “Navigating,” “Cancer Care,” “Oncology,” and “Healthcare.” The goal of this strategic approach was to gather a complete and current corpus of literature on BC and healthcare in the Indian context. Articles, reviews, and reports that directly addressed the intricacies of navigating BC in India were carefully chosen, adhering to predetermined inclusion and exclusion criteria. The inclusion criteria stipulated that the selected materials have to be published in English, address current advancements in BC treatment in the context of Indian healthcare, and cover research done between 2008 and 2023. This thorough search approach was created to enable a thorough examination of the narrative review's content and guarantee an educated investigation of the dynamics of BC in the Indian healthcare environment. The data extracted from these chosen studies were meticulously examined and synthesized to provide a nuanced understanding of the BC landscape in India (Figure 4).

## 3. Effectiveness of Current Screening Programs

A few factors that determine whether screening programs are successful or not include the availability of adequate human resources, the development and use of an appropriate diagnostic tool, appropriate execution, and the presence of adequate instruction manuals [16]. Another factor to consider is how well the screening test works to lower the likelihood of false-positive results and unnecessary biopsies and procedures. Organized screening programs use multidisciplinary delivery teams, coordinated clinical oversight committees, and routine evaluation by a multispecialty assessment board to optimize the benefit to the target population [17]. Additionally, check for particular target demographic is done. Screening methods are becoming more risk based rather than advocating a wide age and sex range. To use this risk-based approach, India needs to assess risk factors and incorporate the findings into BC screening [18].

A recent study conducted in Mumbai found that clinical breast examination (CBE) conducted every 2 years by primary healthcare providers significantly decreased the stage of BC at diagnosis and led to a 15% decrease in the overall death rate from BC (though there was a significant reduction of nearly 30% in mortality in women aged ≥ 50). Mammography has been found to have a sensitivity range of 64%–90% and a specificity range of 82%–93% [19]. The breast density of Indian women is higher, and there are insufficient mammography machines and skilled workers [20]. This could lead to overdiagnosis and false positives. Even with computer-assisted detection technologies, digital mammography remains costly [21]. These factors make frequent mass mammography screening a less desirable option for a developing nation like India.

With an overall sensitivity of 53%–67% and specificity of 89%–99%, ultrasound (US) is a potentially useful diagnostic tool for younger women (ages 40–49) [22]. The requirement that ultrasonography be done and interpreted by trained professionals is one major barrier. Breast self-examination (BSE) is not approved as a method for early BC identification, but when carried out skillfully and with care, it can be a useful tool for educating women about the characteristics of a normal breast. It might be able to identify women who are at a high risk of acquiring cancer and implement personalized screening at a low cost by studying genomes to understand changes unique to India. There is an urgent need to look for genetic and epigenetic markers specific to India. These could be used as biomarkers in the screening process to identify issues early [23].

## 4. Regional Disparities in Screening Uptake

Currently, the Indian government spends 1.6% of its gross domestic product (GDP) on healthcare, meaning that the majority of people must pay for their screening and treatment [24]. As a result, behaviors related to obtaining medical attention are heavily reliant on financial resources, including health literacy and education, which differ according to gender. In India, women spend much less on average for healthcare, and this disparity is even worse when they have serious illnesses like cancer that need extensive care. Women are frequently denied access to life-saving treatments due to a lack of financial assistance, unequal access to financial resources, and the stigma attached to cancer. The issue is further compounded by late-stage diagnosis and unequal access to treatment [25].

Operational Guidelines have been released by the Ministry of Health and Family Welfare for three of the most prevalent malignancies in India: lip/oral, uterine/cervical, and BC. The guidelines seek to improve early detection by conducting community-based screening once every 5 years using electronic health cards and population enumeration. This takes a lot of time and resources to implement. Meanwhile, free mobile screening camps are being provided in areas like Delhi thanks to funding from NGOs and the government. These camps remove significant financial and accessibility hurdles by bringing cancer screening straight to neighborhoods [26]. Between population-based screening and opportunistic screening, mobile screening camps act as a transitional step. In the former approach, factors such as an individual's capacity to pay, awareness, and choice play a major role in determining attendance; in the latter approach, many of these obstacles are lessened. While the population-based strategy has some advantages, the mobile screen camp recruits and screens women with less infrastructure. The varying population variability in terms of access to follow-up care facilities, socioeconomic status, and residence status presents additional obstacles to this strategy [27].

## 5. Screening Modalities and Technologies

Compared to women in the West, Indian women have a lower chance of developing BC. In contrast to 60%–70% of occurrences in the industrialized world, only 30% of cases of BC reported from different parts of India are early cases [28]. The primary cause of the high death rate among these patients is the fact that over 70% of the women present at an advanced stage. The absence of a BC screening program and women's nonparticipation if one was implemented are the two key themes of the entire advanced-stage presentation. The reasons for this include society and cultural norms, ignorance, and restricted access to healthcare. A few essential components are needed for a high-level screening program: high-quality screening, extensive coverage, high participation rates, and a functional system for referrals for diagnosis and treatment. While the United States and the United Kingdom have claimed to have great screening rates, India has nearly nonexistent screening rates [29].

Programs for organized screening have demonstrated a decline in the number of deaths from BC in the West. Currently, BSE, CBE, and mammography are the three methods most frequently used worldwide to assess BC [30]. Mammography and routine CBE can lower the number of deaths from BC by downstaging the disease in asymptomatic women [31]. In this article, we have covered mammography, breast MRI (magnetic resonance imaging), CBEs, and genetic testing for the detection of BC in India. Systemic spread of BC is a risk factor that rises with tumor size, local infiltration, and lymph node metastasis. Certain research is necessary for clinical management, particularly when desired research is not possible for a variety of reasons and is advised against for varying phases of the disease.  Table 2 shows the diagnostic and staging modalities for different BC stages.

### 5.1. Mammography

Using a variety of research techniques, including randomized controlled trials and observational studies (trend analyses, cohort studies, and case–control studies), mammography screening has been linked to a lower death rate from BC. Most of the studies have shown a statistically significant benefit. According to studies, mammography screenings, which should be done every 12–33 months, can dramatically lower the death rate from BC. The majority of the benefits of screening mammography are maintained by biennial screening [33]. Women between the ages of 55 and 64 are more likely to get BC, and the risk rises with age. Over time, this increased risk may make it possible to identify BC at an earlier stage, which would increase the prognosis for the patient and lessen adverse effects from therapy. BC treatments are still developing and have helped to lower mortality, even though early diagnosis through mammography screening has had a greater overall impact [34].

False-positive results from BC screening have been connected to recalling noncancerous women for additional imaging or biopsy [35]. Estimates of overdiagnosis vary widely, ranging from less than 5% to over 50%. A mammography screening program is a complex, comprehensive project. Since the accuracy of mammography screening depends on elements including the requirement for high-quality equipment, trained radiologists, and large resources and infrastructure that are out of reach for most lower–middle-income countries, it is not considered cost-effective for a lower–middle-income country [36].

### 5.2. CBE

In the Canadian National Breast Screening Study 2, comparing CBE with CBE + mammography did not reveal any significant benefits [37]. In its intermediate analysis, the 2006 Trivandrum randomized controlled trial showed early-stage detection of BCs with CBE [38]. The Mumbai randomized controlled trial with CBE, one of the oldest and longest studies with biannual CBE administered four times in one arm against no screening in the other, showed a reduction in BC mortality among women over 50 and downstaging with CBE for all age groups. CBE screening in India may be a practical, cost-effective, and helpful option in lower–middle-income countries [39]. If healthcare professionals are not trained in CBE and are not used in early detection programs, women in low- and middle-income countries might not have the chance to frequently undergo breast exams for early BC diagnosis. As a screening and diagnostic tool, CBE is crucial for isolated and rural areas that have little access to costly technology resources [40].

### 5.3. Breast MRI

Since its widespread usage in 2000, breast MRI has emerged as a crucial tool for the detection, diagnosis, staging, and follow-up of high-risk BC. Breast MRI is useful for several purposes, including high-risk screening; assessing unknown primary origin; assessing local disease extent, multicentricity, and bilaterally, particularly in dense breasts; separating a scar from a local recurrence in women who have undergone breast-conserving surgery; assessing response to neoadjuvant chemotherapy; and assessing implant integrity. But there are a lot of debates about breast MRIs [41]. To view the anatomy of the breasts or the internal mammary glands, a breast MRI is obtained. It operates by taking inside breast images with radio waves and magnets. Mammography and breast MRI are typically combined, particularly in high-risk situations [42]. Images of the interior of the breast are obtained using an MRI. It uses a computer, powerful magnets, and radio waves to produce incredibly detailed images. A biopsy may be performed if it indicates malignancy. An MRI of the breast can identify issues with the other breast in addition to the location of malignancy [43].

There are significant differences in practice when it comes to the contentious use of breast MRI in the preoperative phase for women who have recently been diagnosed with BC. Preoperative MRI can identify multifocal and multicentric lesions and assess the contralateral breast, particularly in cases of lobular carcinoma and dense breast tissue [44]. When it comes to women who are at average risk, MRI screening has not been very popular because of worries about its limited specificity, which can result in unnecessary biopsies, time, and expense for equipment. It is also clear that women with thick breasts have restricted access to mammography due to its reduced sensitivity. Additionally, mammography is said to detect more slow-growing tumors. Breast MRI employs intravenous contrast (gadolinium) and preferentially detects higher-grade lesions, not restricted by breast density [41].

### 5.4. Genetic Testing

Saliva, blood, and other noncancerous tissues are the major subjects of genetic testing for BC to identify inherited genetic changes from your parents that increase your risk of developing BC. Breast MRI, mammography, and genetic testing are the three major breast screening tests that, when carried out under the supervision of a qualified physician, are advised for women over the age of 50. You should see your doctor as soon as possible if you have observed any of the symptoms [45]. Mittal et al. [46] have conducted research. In their research, overall, 275 BC patients were screened, and 236 patients were included (median age 45 years); 30 patients did not consent, and 9 patients previously underwent genetic testing. Thirty-four (14%) women had a positive family history and 35% had TNBC. P/LP mutations were found in 44/236 (18.64%) women; they found mutations in BRCA1 (22/47, 46.8%) and BRCA2 (9/47, 19.1%) were the most common, with 34% of mutations present in non-BRCA genes. In conclusion, the frequency of P/LP mutations in India is high, with a significant contribution of non-BRCA genes. Testing criteria need modification to expand access to testing.

In Western literature, germline pathogenic mutations have been linked to 10–15% of BC cases, with BRCA1/2 responsible for 40%–50% of hereditary BC cases [47]. Mutations in additional BC-causing genes, such as PALB2, ATM, CHEK, and TP53, are becoming more frequently found as multigene panel testing becomes more widely used. In comparison to the West, the Indian scenario has a lower incidence-to-mortality ratio due to a higher prevalence of TNBC (up to 30%) and a younger median age of onset (< 50 years). With these factors, along with delayed access to effective BC screening programs and limited access to treatment [48, 49], India is predicted to have a greater population incidence of BRCA mutations since these mutations are more common in the younger TNBC population [50]. There are limited centers for comprehensive genetic counseling, genetic testing, and medical management of carriers in India.  Table 3 depicts the comparison of screening modalities and technologies.

## 6. Artificial Intelligence (AI) Technologies for BC Screening

### 6.1. iBreastExam (iBE)

“iBE” is a “portable, clinically proven, noninvasive, painless, and radiation-free instrument” that was created by UE Lifesciences to aid in the early detection of breast lesions at the site of contact. The breast exam also called the iBE is made up of three parts: a tablet, a specially designed electrical board, and a portable compression probe with a 4 × 4 array of piezoelectric tactile pressure sensors. To see and store data in real time, the iBE establishes a wireless connection with a mobile device. After every scan, the compression results are stored separately on the mobile device and synchronized with an encrypted database via Dropbox, where a current duplicate is kept up to date. Anonymity is preserved because the iBE program does not gather or save personally identifying information [54].

The lead investigator of the trial and the maker of the device have trained an ultrasound technician to do iBE evaluations. The training included an hour-long lecture on the device's operations, 10 iBE studies under supervision, and practice with a breast phantom. The results were shown on a touch screen iBE pressure map. Normal breast tissue is shown by green, whereas the presence of a lesion is indicated by red. Since the iBE is unable to distinguish between various types of lesions, further testing is advised to fully characterize the lesion. During the examination, the patient is in a supine position [55]. The gadget may need to be calibrated multiple times on an unaffected breast area. Each square in the 4 × 4 array map of the breast generated by the iBE represents the 4 × 4 array of the piezoelectric finger sensor (PEFS). The outcome of compiling the gathered data is this map. To enable a direct comparison with the clock positions assigned by mammography or ultrasonography, this map was divided into sectors defined by three consecutive hours of a clock [54].

### 6.2. Computer-Aided Detection (CADe)

In the field of radiology, computer algorithm advancements are getting more complex and pervasive. These advancements have the potential to save costs by raising the detection rates of various medical disorders and enhancing radiologists' productivity [56]. CADe is a method that machine learning has used in breast imaging [57]. CADe identifies suspicious regions of possible cancer characteristics, such as masses and microcalcifications, in digital mammograms. After completing their interpretations, radiologists typically evaluate these markings and compare the two to determine the image's final evaluation [58].

The main objective of CADe systems is to identify anomalous symptoms that a specialist would overlook and to offer more accurate diagnostic evaluations as a second opinion to counter the specialists' subjective interpretation [59]. The use of CADe systems is limited to the identification of anomalous constructions or pieces. Conversely, computer-aided diagnostics (CADx) evaluates the aberrant regions found in CADe to offer diagnostic options. The combined use of the CADe and CADx models is crucial for early anomaly detection. Over the past 40 years, numerous CADe algorithms have been developed employing different image modalities, including x-ray, MRI, and US, for the early detection of BC [60]. In CADe systems, histology pictures are also utilized. Microcalcification clusters, tiny lumps in dense tissue, the marginal structure of suspicious masses, architectural distortions, and the extremely dense tissue structure in mammograms are all recognized and highlighted by x-ray–based CADe systems. Additionally, the CADe systems can distinguish between a benign and malignant mass based on features like size, shape, and texture, making it simple for radiologists to make diagnoses. In single-view systems, the mediolateral oblique (MLO) or craniocaudal (CC) views are employed. Double-view systems combine the two views to make use of the information that was overlooked in the first view [61].

For screening purposes, mammography-based CADe is typically recommended since it can identify breast abnormalities before the development of symptoms. Using powerful radio waves and magnetic waves, MRI creates exquisite details of the tissues inside the breasts and is very useful in identifying high-risk BC cases. Sound waves can be used to create an elastogram or echogram, which is the breast US [59]. While the elastogram replicates the elasticity of the identical tumor areas, echography reconstructs their echo reflection property. Both might be utilized to create a CADe and offer a second opinion on the diagnosis. There are not many CADe solutions that combine the elastographic and echographic characteristics of these ultrasonography pictures to produce more insightful results. For individuals at high risk who are unable to receive MRIs or pregnant women to whom x-ray exposure should be avoided, the US of the breast is recommended. Additionally, the US is a very popular screening technique for women whose breast tissues are denser than what an x-ray can detect [62].

### 6.3. Automated Breast Ultrasound (ABUS)

Mammography screening has less sensitivity in women with thick breasts. Compared to those with fatty breasts, the chance of developing BC is seven to eight times higher in those with dense glandular tissue. For better cancer detection, more imaging modalities are therefore needed. The Food and Drug Administration (FDA) approved ABUS in 2012 as an additional screening tool for women with exceptionally thick and diverse breasts [63]. ABUS is a technique that reduces operator dependence and physician time by separating the moment of image interpretation (made by the radiographer) from the moment of picture acquisition. Coronal reconstructions also provide fresh diagnostic data. Thus, this method was created to standardize breast US and get rid of some of the drawbacks of hand-held ultrasound (HHUS), like operator dependence and examination duration [64].

ABUS is an US technique that is gaining popularity as an adjuvant in the evaluation of patients with dense glandular breasts. Patients with dense breasts have a higher risk of developing BC in comparison to those with fatty breasts. Furthermore, in this patient population, mammography is not very sensitive in detecting BC, especially if there are no calcifications or architectural abnormalities. There are many advantages to using the ABUS standardized test for screening and diagnosis. It expedites workflow, shortens exam times, and increases the number of BC diagnoses [65]. Given that the images are obtained by nonmedical staff, ABUS offers several benefits, including low operator dependency and excellent repeatability. The ability to save images on a specialized station facilitates multiplanar reconstructions, double reading, and objective comparison with earlier analyses [66]. Reproducible images for breast lesion location, size measurement, and characterization are provided by ABUS, which has been helpful, particularly in clinical scenarios where follow-up imaging is necessary [63].

## 7. Barriers to Timely Screening

Many wealthy countries have developed screening programs for BC. Developing countries such as India have not had these screening priorities. As evidenced by the fact that half of the participants in our survey were ignorant of BC, women need to be educated about health issues. Indian primary healthcare practitioners are knowledgeable about the early detection of BC and the risk factors that contribute to the disease. Through the implementation of early detection campaigns, awareness programs, and treatments that minimize the burden of illness, these frontline healthcare personnel may improve cancer literacy [67].

The ignorance of BC was one of the challenges brought up by the female participants. This result can be explained by the low rate of literacy and poverty among women. The lady and occasionally her family members interpret the symptoms differently, so even in cases when there are noticeable clinical signs such as lymph node enlargement, redness, and localized swelling, the lack of understanding about the condition causes delays in seeking medical assistance. One of the main societal barriers to early diagnosis was also the dread of cancer, both in terms of detection and treatment. Delays in diagnosis are caused by a lack of health resources, which has been a major issue elsewhere as well. There are differences in the accessibility of health facilities among the various Indian states. Low levels of BC screening have been linked to low socioeconomic status, lack of a regular source of healthcare, and fear of the disease in underdeveloped nations. For some women, the screening process itself was a personal barrier since they were terrified of learning they had cancer [68].

## 8. Patient Education and Awareness

In India, patient awareness and education are crucial in the battle against BC. It is empowering for people to know the fundamentals of BC, its risk factors, and the value of early detection. It is important to educate women about routine screenings, such as CBEs and mammograms, to identify possible problems early on and treat them more effectively. It is crucial to promote BSE as a regular component of healthcare procedures since it gives people a sense of agency over their health. A holistic strategy for the treatment of BC benefits from easily accessible information about available treatment options, emotional support, and follow-up care. Making sure that these essential tools are understood and resonate with India's diverse people requires careful consideration of cultural differences and good communication. Our goal is to enable women to prioritize their breast health by raising awareness and educating others, so developing a proactive and informed approach to BC prevention and care is imperative [69].

## 9. Conclusion

The overall review's conclusion emphasizes how urgent it is to develop novel screening techniques in order to combat India's escalating BC incidence. We suggest a comprehensive strategy that makes use of cutting-edge technologies like portable breast exams and AI-driven diagnostics, as well as customized risk-stratified screening methods. Furthermore, we stress the significance of programs such as mobile screening camps in reducing expenses and obstacles related to accessibility. The evaluation also emphasizes the necessity of providing readers and physicians with information and practical next steps. In the future, it will be critical to promote the use of these screening techniques in clinical settings, stressing the value of early detection and patient education. It is recommended that clinicians give priority to integrating risk-stratified screening protocols into their practice and maintaining current knowledge of evolving recommendations and technology. Also, a proactive approach to BC prevention and care in India must address cultural taboos surrounding breast health and support awareness initiatives. By providing this information to the public, legislators, and healthcare professionals, we can spur joint efforts to improve outcomes and lower the incidence of BC nationwide. To put it briefly, the following steps entail putting this knowledge into practice through the implementation of risk-stratified screening procedures, patient education and awareness campaigns, and the removal of cultural barriers related to breast health. To make a real difference in BC outcomes in India, tailored interventions, financial allocation, and community engagement are needed.

## Figures and Tables

**Table 1:** Breast cancer statistics by state in India [11, 12].

**State**	**Estimated new cases (2020)**	**Estimated deaths (2020)**	**Age–standardized incidence rate (per 100,000)**
Maharashtra	35,963	15,309	29.1
Tamil Nadu	29,170	12,405	29.4
West Bengal	23,459	9,901	22.0
Andhra Pradesh	22,916	9,838	20.2
Karnataka	21,940	9143	22.6
Uttar Pradesh	19,782	8143	15.7
Gujarat	16,176	6705	17.7
Kerala	15,913	6699	24.3
Telangana	14,705	6205	19.8
Madhya Pradesh	14,350	5926	16.1

**Table 2:** Diagnostic and staging modalities for different breast cancer stages: A comparative overview. Adapted with permission from [32], copyright, 2016 ICMR.

	**Operable breast cancer (OBC)**	**Locally advanced breast cancer (LOBC/LABC)**	**Metastatic breast cancer (MBC)**	**Purpose/comments**
Routine tests (CBC, biochemistry)	Yes	Yes	Yes	To assess fitness for anesthesia and chemotherapy

Breast imaging	Yes	Yes	In selected cases where it is clinically indicated	B/L mammography: If the breast lump is suspected to be malignant, especially if BCT is being considered USG: If cystic/benign lesion is suspected, especially in young women MRI: In expert centers, breast MRI is useful in screening or characterizing breast lumps if mammography is suboptimal due to dense breast (as in some young women) or prior breast reconstruction, especially useful for screening young women at high risk of developing breast cancer due to family history or BRCA mutation

Cytological/histopathological confirmation of diagnosis	Yes	Yes	Yes	Core biopsy: Preferred method in all cases and mandatory if neoadjuvant systemic therapy is planned for histological grading and receptor status. To mark the site of the primary tumor, the core biopsy should be centered over the tumor FNAC: Acceptable in cases with clinical and mammographically evident cancer planned for upfront surgery Incision or excision biopsy: When there is high clinical suspicion and repeated FNAC/core biopsy is negative

ER/PR	Yes	Yes	Yes	IHC (> 1% tumor cells staining for ER considered ER+ve)

HER2	Yes	Yes	Yes	More relevant in cases for whom trastuzumab is feasible standardized IHC for HER2; if IHC is equivocal (2+), then FISH

Chest x-ray	Yes	Yes	Yes	To assess fitness for anesthesia and for staging in LOBC/LABC

Bone scan	No	Yes	Yes	If raised alkaline phosphatase or bone symptoms/signs. If a bone scan is not feasible due to various logistical or healthcare provision issues, perform a skeletal survey, especially if symptomatic

USG abdomen	No	Yes	Yes	If abnormal LFT or suspicious symptoms/signs. Not required if CT thorax and abdomen is being performed

CT thorax/abdomen	No	Yes	Yes	If abnormal LFT or suspicious symptoms/signs. If a CT scan is not feasible due to various logistical or healthcare provision issues, chest x-ray and USG abdomen for staging

FDG PET/CT scan	No	In selected cases	In selected cases	Especially useful if standard imaging findings are equivocal

Tumor markers (CA-15.3, etc.)	No	No	No	Clinical utility in making diagnosis and disease monitoring not yet established

Multigene signature MammaPrint and Oncotype Dx	No	Not applicable	Not applicable	Their clinical utility and added value in routine practice are as yet unknown and there is very little data in Indian patients

**Table 3:** Comparison of screening modalities and technologies.

**Serial no.**	**Screening modernity/technology**	**Description**	**Advantages**	**Limitations**	**References**
1	Mammography	Overview of mammography for breast cancer screening	Early diagnosis and prevention of late-stage presentation of disease	Expensiveness and cumbersomeness	[51]

2	CBE	Physical examination of the breasts performed by a trained healthcare provider	Noninvasive Low cost Potential for early detection	False positives with additional testing and anxiety False negatives with potential false reassurance and delay in cancer diagnosis	[52]

3	Breast MRI	Used as a supplemental tool to breast screening with mammography or ultrasound	High sensitivity Useful for high-risk individuals	Costly Limited availability	[41]

4	Genetic testing	Medical test that examines a person's DNA to identify changes or mutations in their genes	A sense of relief from uncertainty Reduce the risk of cancer by making certain lifestyle changes if the patient has a positive result In-depth knowledge about your cancer risk Information to help make informed medical and lifestyle decisions	Costly Limited access to genetic counseling Testing may increase anxiety and stress for some individuals Testing does not eliminate a person's risk for cancer	[53]

## Data Availability

The data analyzed in this study were sourced from publicly available articles in the open literature. All references to the data used, including article titles, authors, and publication sources, are provided in the reference list of this article. Readers and researchers interested in accessing the raw data can refer to the original publications cited herein for retrieval from the respective sources.

## Nomenclature

ABUS: automated breast ultrasound
AI: artificial intelligence
BC: breast cancer
BSE: breast self-examination
CADe: computer-aided detection
CADx: computer-aided diagnostics
CBE: clinical breast examination
FDA: Food and Drug Administration
GDP: gross domestic product
Her2: human epidermal growth factor receptor 2
HHUS: hand-held ultrasound
iBE: iBreastExam
MRI: magnetic resonance imaging
PEFS: piezoelectric finger sensor
TNBC: triple-negative breast cancer
US: ultrasound

## References

[1] Sung H., Ferlay J., Siegel R. L. (2021). Cancer statistics 2020: GLOBOCAN estimates of incidence and mortality worldwide for 36 cancers in 185 countries. *CA: A Cancer Journal for Clinicians*.

[2] Hospital GS (2023). *Early Detection Matters: Navigating Breast Cancer Screening in India. Medium*.

[3] Mehrotra R., Yadav K. (2022). Breast cancer in India: present scenario and the challenges ahead. *World Journal of Clinical Oncology*.

[4] Dhillon P. K., Mathur P., Nandakumar A. (2018). The burden of cancers and their variations across the states of India: the Global Burden of Disease Study 1990–2016. *The Lancet Oncology*.

[5] International Agency for Research on Cancer Breast cancer factsheet. Global cancer observatory. https://gco.iarc.fr/today/data/factsheets/cancers/20-Breast-fact-sheet.pdf.

[6] Breast Cancer India Latest statistics of breast cancer in India 2020. https://www.breastcancerindia.net/statistics/latest_statistics_breast_cancer_india.html.

[7] Malvia S., Bagadi S. A., Dubey U. S., Saxena S. (2017). Epidemiology of breast cancer in Indian women. *Asia-Pacific Journal of Clinical Oncology*.

[8] Arumugham R., Raj A., Nagarajan M., Vijilakshmi R. (2014). Survival analysis of breast cancer patients treated at a tertiary care centre in Southern India. *Annals of Oncology*.

[9] Maurya A. P., Brahmachari S. (2021). Current status of breast cancer management in India. *The Indian Journal of Surgery*.

[10] IARC Publications Website World cancer reports. https://publications.iarc.fr/Non-Series-Publications/World-Cancer-Reports.

[11] Mathur P., Sathishkumar K., Chaturvedi M. (2020). Cancer statistics, 2020: report from National Cancer Registry Programme, India. *JCO Global Oncology*.

[12] Population Based Cancer Registry ICMR–National institute of cancer prevention and research. https://nicpr.org/population-based-cancer-registry.

[13] NCI (2023). Breast cancer screening. https://www.cancer.gov/types/breast/patient/breast-screening-pdq.

[14] Early cancer diagnosis saves lives, cuts treatment costs. https://www.who.int/news/item/03-02-2017-early-cancer-diagnosis-saves-lives-cuts-treatment-costs.

[15] Breast cancer. https://www.who.int/news-room/fact-sheets/detail/breast-cancer.

[16] Rajaraman P., Anderson B. O., Basu P. (2015). Recommendations for screening and early detection of common cancers in India. *The Lancet Oncology*.

[17] Sivaram S., Majumdar G., Perin D. (2018). Population-based cancer screening programmes in low-income and middle-income countries: regional consultation of the International Cancer Screening Network in India. *The Lancet Oncology*.

[18] El Saghir N. S., Charara R. N. (2014). International screening and early detection of breast cancer: resource-sensitive, age- and risk-specific guidelines. *Breast Cancer Management*.

[19] Mittra I., Mishra G. A., Dikshit R. P. (2021). Effect of screening by clinical breast examination on breast cancer incidence and mortality after 20 years: prospective, cluster randomised controlled trial in Mumbai. *BMJ*.

[20] Singh T., Khandelwal N., Singla V. (2018). Breast density in screening mammography in Indian population - is it different from western population?. *The Breast Journal*.

[21] Fuller M. S., Lee C. I., Elmore J. G. (2015). Breast cancer screening: an evidence-based update. *Medical Clinics*.

[22] Li J., Shao Z. (2015). Mammography screening in less developed countries. *Springerplus*.

[23] Gupta R., Gupta S., Mehrotra R., Sodhani P. (2020). Risk factors of breast cancer and breast self-examination in early detection: systematic review of awareness among Indian women in community and health care professionals. *Journal of Public Health*.

[24] IBEF India Brand Equity Found Indian healthcare industry analysis. https://www.ibef.org/industry/healthcare-presentation.

[25] Saikia N., Moradhvaj, Bora J. K. (2016). Gender difference in health-care expenditure: evidence from India human development survey. *PloS One*.

[26] Mehrotra R., Yadav K. (2022). Cervical cancer: formulation and implementation of Govt of India guidelines for screening and management. *Indian Journal of Gynecologic Oncology*.

[27] Sekar P., Ghosh S., Dhillon P., Shridhar K. (2022). The dynamics of breast cancer screening approaches in urban India: an ethnographic study from Delhi. *SSM-Qualitative Research in Health*.

[28] PubMed Awareness of breast cancer in women of an urban resettlement colony. https://pubmed.ncbi.nlm.nih.gov/19112202/.

[29] Singh S., Shrivastava J. P., Dwivedi A. (2015). Breast cancer screening existence in India: a nonexisting reality. *Indian Journal of Medical and Paediatric Oncology*.

[30] Takkar N., Kochhar S., Garg P., Pandey A. K., Dalal U. R., Handa U. (2017). Screening methods (clinical breast examination and mammography) to detect breast cancer in women aged 40–49 years. *Journal of Mid-Life Health*.

[31] Ginsburg O., Yip C. H., Brooks A. (2020). Breast cancer early detection: a phased approach to implementation. *Cancer*.

[32] https://speciality.medicaldialogues.in/icmr-guidelines-on-how-to-diagnose-stage-and-evaluate-workup-for-breast-cancer.

[33] Oeffinger K. C., Fontham E. T., Etzioni R. (2015). Breast cancer screening for women at average risk: 2015 guideline update from the American Cancer Society. *JAMA*.

[34] Reeves R. A., Kaufman T. (2024). Mammography. *Stat Pearls*.

[35] Grimm L. J., Avery C. S., Hendrick E., Baker J. A. (2022). Benefits and risks of mammography screening in women ages 40 to 49 years. *Journal of Primary Care and Community Health*.

[36] Chaltiel D., Hill C. (2021). Estimations of overdiagnosis in breast cancer screening vary between 0% and over 50%: why?. *BMJ Open*.

[37] Chopra S., Khosla M., Vidya R. (2023). Innovations and challenges in breast cancer care: a review. *Medicina*.

[38] Ramadas K., Basu P., Mathew B. S. (2023). Effectiveness of triennial screening with clinical breast examination: 14-years follow-up outcomes of randomized clinical trial in Trivandrum, India. *Cancer*.

[39] Ngan T. T., Nguyen N. T. Q., Minh H. V., Donnelly M., O’Neill C. (2020). Effectiveness of clinical breast examination as a ‘stand-alone’ screening modality: an overview of systematic reviews. *BMC Cancer*.

[40] Veitch D., Goossens R., Owen H., Veitch J., Molenbroek J., Bochner M. (2019). Evaluation of conventional training in clinical breast examination (CBE). *Work*.

[41] Radhakrishna S., Agarwal S., Parikh P. M. (2018). Role of magnetic resonance imaging in breast cancer management. *South Asian Journal of Cancer*.

[42] (2021). Breast magnetic resonance imaging (MRI). https://www.hopkinsmedicine.org/health/treatment-tests-and-therapies/breast-mri.

[43] Bougias H., Stogiannos N. (2022). Breast MRI: where are we currently standing?. *Journal of Medical Imaging and Radiation Sciences*.

[44] Chan W. Y., Cheah W. K., Ramli Hamid M. T. (2022). Impact of preoperative magnetic resonance imaging on surgery and eligibility for intraoperative radiotherapy in early breast cancer. *PLoS One*.

[45] Lynch J., Venne V., Berse B. (2015). Genetic tests to identify risk for breast cancer. *Seminars in Oncology Nursing*.

[46] Mittal A., Deo S. V. S., Gogia A. (2022). Profile of pathogenic mutations and evaluation of germline genetic testing criteria in consecutive breast cancer patients treated at a north Indian tertiary care center. *Annals of Surgical Oncology*.

[47] Mehrgou A., Akouchekian M. (2016). The importance of BRCA1 and BRCA2 genes mutations in breast cancer development. *Medical Journal of the Islamic Republic of Iran*.

[48] Han M. R., Zheng W., Cai Q. (2017). Evaluating genetic variants associated with breast cancer risk in high and moderate-penetrance genes in Asians. *Carcinogenesis*.

[49] Sandhu G. S., Erqou S., Patterson H., Mathew A. (2016). Prevalence of triple-negative breast cancer in India: systematic review and meta-analysis. *Journal of Global Oncology*.

[50] Engel C., Rhiem K., Hahnen E. (2018). Prevalence of pathogenic *BRCA1/2* germline mutations among 802 women with unilateral triple-negative breast cancer without family cancer history. *BMC Cancer*.

[51] Weerarathna I. N., Kamble A. R., Luharia A. (2023). Artificial intelligence applications for biomedical cancer research: a review. *Cureus*.

[52] Raghavan N., Jatoi I. (2024). Prioritizing mammography screening in developing countries: are we putting the cart before the horse?. *Annals of Surgical Oncology*.

[53] Rummel S. K., Lovejoy L. A., Turner C. E., Shriver C. D., Ellsworth R. E. (2020). Should genetic testing for cancer predisposition be standard-of-care for women with invasive breast cancer? The murtha cancer center experience. *Cancers*.

[54] Bhattacharya S., Varshney S., Heidler P., Tripathi S. K. (2022). Expanding the horizon for breast cancer screening in India through artificial intelligent technologies-a mini-review. *Frontiers in Digital Health*.

[55] Browne J. E., Cannon L. M., McDermott R., Ryan M., Fagan A. J. (2017). Pilot investigation into the use of an anthropomorphic breast sonography phantom as a training and assessment tool. *Ultrasound in Medicine & Biology*.

[56] Dreyer K. J., Geis J. R. (2017). When machines think: radiology’s next frontier. *Radiology*.

[57] Masud R., Al-Rei M., Lokker C. (2019). Computer-aided detection for breast cancer screening in clinical settings: scoping review. *JMIR Medical Informatics*.

[58] Keen J. D., Keen J. M., Keen J. E. (2018). Utilization of computer-aided detection for digital screening mammography in the United States, 2008 to 2016. *Journal of the American College of Radiology*.

[59] Arun Kumar S., Sasikala S. (2023). Review on deep learning-based CAD systems for breast cancer diagnosis. *Technology in Cancer Research & Treatment*.

[60] Firmino M., Angelo G., Morais H., Dantas M. R., Valentim R. (2016). Computer-aided detection (CADe) and diagnosis (CADx) system for lung cancer with likelihood of malignancy. *Biomedical Engineering Online*.

[61] Ramadan S. Z. (2020). Methods used in computer-aided diagnosis for breast cancer detection using mammograms: a review. *Journal of Healthcare Engineering*.

[62] Sigrist R. M. S., Liau J., Kaffas A. E., Chammas M. C., Willmann J. K. (2017). Ultrasound elastography: review of techniques and clinical applications. *Theranostics*.

[63] Boca I., Ciurea A. I., Ciortea C. A., Dudea S. M. (2021). Pros and cons for automated breast ultrasound (ABUS): a narrative review. *Journal of Personalized Medicine*.

[64] Łuczyńska E., Pawlak M., Popiela T., Rudnicki W. (2022). The role of ABUS in the diagnosis of breast cancer. *Journal of Ultrasonography*.

[65] Ali E. A., Ahmed A. M., Elsaid N. A. (2020). The added advantage of automated breast ultrasound to mammographically detected different breast lesions in patients with dense breasts. *Egyptian Journal of Radiology and Nuclear Medicine*.

[66] Zanotel M., Bednarova I., Londero V. (2018). Automated breast ultrasound: basic principles and emerging clinical applications. *La Radiologia Medica*.

[67] D’almeida D., Latha T. (2021). Barriers for early detection of breast cancer among south Indian women. *Indian Journal of Community Medicine*.

[68] Gupta A., Shridhar K., Dhillon P. K. (2015). A review of breast cancer awareness among women in India: cancer literate or awareness deficit?. *European Journal of Cancer*.

[69] Madhukumar S., Thambiran U. R., Basavaraju B., Bedadala M. R. (2017). A study on awareness about breast carcinoma and practice of breast self-examination among basic sciences’ college students, Bengaluru. *Journal of Family Medicine and Primary Care*.

